# Cooperative evolution of two different TEs results in lineage-specific novel transcripts in the *BLOC1S2* gene

**DOI:** 10.1186/s12862-019-1530-0

**Published:** 2019-10-30

**Authors:** Hyeon-Mu Cho, Sang-Je Park, Se-Hee Choe, Ja-Rang Lee, Sun-Uk Kim, Yeung-Bae Jin, Ji-Su Kim, Sang-Rae Lee, Young-Hyun Kim, Jae-Won Huh

**Affiliations:** 1 National Primate Research Center, Korea Research Institute of Bioscience and Biotechnology (KRIBB), Cheongju, 28116 Korea; 2 Department of Functional Genomics, KRIBB School of Bioscience, Korea University of Science & Technology (UST), Daejeon, 34113 Korea; 3 Primate Resource Center, Korea Research Institute of Bioscience and Biotechnology (KRIBB), Jeongeup, 56216 Korea; 4 Futuristic Animal Resource and Research Center, Korea Research Institute of Bioscience and Biotechnology (KRIBB), Cheongju, 28116 Korea

**Keywords:** Primate, Transposable element, Sequential integration, Alternative splicing, Branch point

## Abstract

**Background:**

The *BLOC1S2* gene encodes the multifunctional protein BLOS2, a shared subunit of two lysosomal trafficking complexes: i) biogenesis of lysosome-related organelles complex-1 and i) BLOC-1-related complex. In our previous study, we identified an intriguing unreported transcript of the *BLOC1S2* gene that has a novel exon derived from two transposable elements (TEs), MIR and *Alu*Sp. To investigate the evolutionary footprint and molecular mechanism of action of this transcript, we performed PCR and RT-PCR experiments and sequencing analyses using genomic DNA and RNA samples from humans and various non-human primates.

**Results:**

The results showed that the MIR element had integrated into the genome of our common ancestor, specifically in the *BLOC1S2* gene region, before the radiation of all primate lineages and that the *Alu*Sp element had integrated into the genome of our common ancestor, fortunately in the middle of the MIR sequences, after the divergence of Old World monkeys and New World monkeys. The combined MIR and *Alu*Sp sequences provide a 3′ splice site (AG) and 5′ splice site (GT), respectively, and generate the Old World monkey-specific transcripts. Moreover, branch point sequences for the intron removal process are provided by the MIR and *Alu*Sp combination.

**Conclusions:**

We show for the first time that sequential integration into the same location and sequence divergence events of two different TEs generated lineage-specific transcripts through sequence collaboration during primate evolution.

## Background

*BLOC1S2*, located on human chromosome 10q24.31, encodes the multifunctional protein BLOS2 that is a shared subunit of two lysosomal trafficking complexes. The first is biogenesis of lysosome- related organelles complex 1 (BLOC-1), which functions in the generation of specialized organelles in the endosomal-lysosomal system, such as platelet dense granules or melanosomes. The other is BLOC-1-related complex (BORC), which has been reported to regulate the positioning of lysosomes [1, 2]. In addition, BLOS2 is involved in several cellular processes independent of BLOC-1 and BORC [1], namely i) the inhibition of the transcriptional suppression activity of BRD7, which is a tumor suppressor candidate [3]; ii) negative regulation of Notch signaling, which is a highly conserved cell-to-cell signaling pathway [1]; and iii) the regulation of apoptotic cell death as the result of interacting with HIPPI (HIP-1 protein interactor) [1, 2]. In addition, BLOS2 is widely expressed both in normal tissue and malignant tumors with a tendency towards lower expression levels in certain tumor subtypes [2].

Alternative splicing (AS) is a post-transcriptional process that contributes significantly to eukaryotic gene diversity by creating multiple isoforms without increasing the genome size [4, 5]. AS is performed by a spliceosome coupled with splicing regulators that bind to cis-acting elements of the target pre-mRNA [6]. The AS mechanism is classified into several types: exon skipping (cassette exons), alternative 5′-splice site (5′-SS), alternative 3′-splice site (3′- SS), intron retention, mutually exclusive exons, alternative promoter, and alternative polyadenylation [4, 7–9]. In the evolutional view, an alternatively spliced exon can be generated by three different molecular mechanisms: exon shuffling, transition of a constitutive exon to an alternative exon, and exonization of intronic sequences [10–12].

Particularly, exonization exploits repetitive mobile elements called transposable elements (TEs) for the event itself. Of the 3 billion base pairs in the human genome, about 45% are made up of TEs, and about 4% of human genes contain TE motifs in their coding region, which suggests an exonization event [8, 12]. TEs are divided into several types, such as DNA transposons (3%), long interspersed nuclear elements (LINEs, 21%), short interspersed nuclear elements (SINEs, 13%), and human endogenous retroviruses (HERVs, 8%) [13–15]. Specifically, *Alu* and MIR, which belong to the SINE family, are known to contribute to alternative splicing [16]. The *Alu* element, the most abundant TE in the human genome, is primate specific and composed of two similar monomers, the left and right arms, and these arms in the antisense orientation provide potential 5′- and 3′-splicing sites that can be recognized by the spliceosome [17, 18]. Moreover, a previous study has shown that *Alu* creates ~ 5% of the alternatively spliced exons in the human genome; thus, it is considered crucial for AS events [19]. MIR is the second most common SINE in primates, representing ~ 2.5% of the human genome, and is interspersed within the mammalian genome [20, 21]. It also provides possible splicing sites for the inserted loci in the antisense orientation [16]. Therefore, TE- derived AS events are crucial sources for multi-transcript creation.

The aims of the present study were the identification and molecular characterization of MIR_*Alu*Sp-derived exonization events in the *BLOC1S2* gene from the evolutionary point of view. Accordingly, we analyzed the approximate integration time of both the MIR and *Alu* elements into the *BLOC1S2* gene using sequencing methods and conducted comparative analyses of the expression patterns of the exonized target sequence in various tissues from rhesus monkey (*Macaca mulatta*), crab-eating monkey (*Macaca fascicularis*), and humans (*Homo sapiens*) to closely examine their interspecies differences. Consequently, this study determined the mechanism by which these two different TEs combined and were involved in the lineage-specific AS event during primate evolution.

## Results

### Comparative analysis of the structure of the *BLOC1S2* gene in humans and non-human primates

**Fig. 1:**
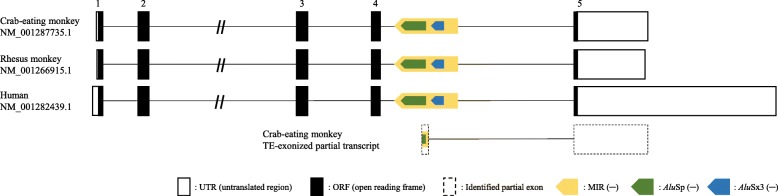
Structural analysis of *BLOC1S2* of the crab-eating monkey, rhesus monkey, and human. The MIR and *Alu*Sp are located in the antisense orientation on the 4th intron of *BLOC1S2*. Open, closed, and dashed line boxes represent the untranslated region of the exon, coding sequence, and identified partial exon, respectively. Yellow, green, and blue arrow boxes represent MIR, *Alu*Sp, and *Alu*Sx3, respectively. This figure is a structural illustration and is not drawn to scale

In a previous study, a large-scale transcriptome sequencing analysis of the genome of the crab-eating monkey enabled us to detect a new partial exon, which is located on the 4th intron of the *BLOC1S2* gene, overlapping sequences of two TEs, *Alu*Sp, and MIR (Fig. 1) [22]. No transcript variants including this partial exon exist in the UCSC Genome Browser database for the crab-eating monkey, rhesus monkey, and human. Prior to identification and experimental validation of this partial exon, we investigated the *BLOC1S2* mRNA sequence of the crab-eating monkey and analyzed its structure comparatively with that of the rhesus monkey and human mRNA sequences. The crab-eating monkey *BLOC1S2* gene transcript (NM_001287735.1) is composed of five exons and transcribes into a mRNA of 1208 bp containing a 19-bp 5′-untranslated region (UTR), a 429-bp coding sequence (CDS), and a 760-bp 3′-UTR. The same conserved five exons also constitute the *BLOC1S2* gene transcripts of both the rhesus monkey (NM_001266915.1) and human (NM_001282439.1), which transcribe into mRNAs of 1158 bp and 2663 bp, respectively (Additional file 1: Figure S1). The notable difference among these three transcripts is the relatively shorter length of the UTRs in the rhesus monkey and crab-eating monkey compared to that of the human UTR. Further, the coding sequence identity was calculated using NCBI Blast, revealing that the *BLOC1S2* gene transcripts of the crab-eating monkey and rhesus monkey were 99% identical and that both were 96% identical to the human *BLOC1S2* gene transcript.

### Evolutionary analysis of the MIR and *Alu*Sp integration event in the *BLOC1S2* gene

**Fig. 2:**
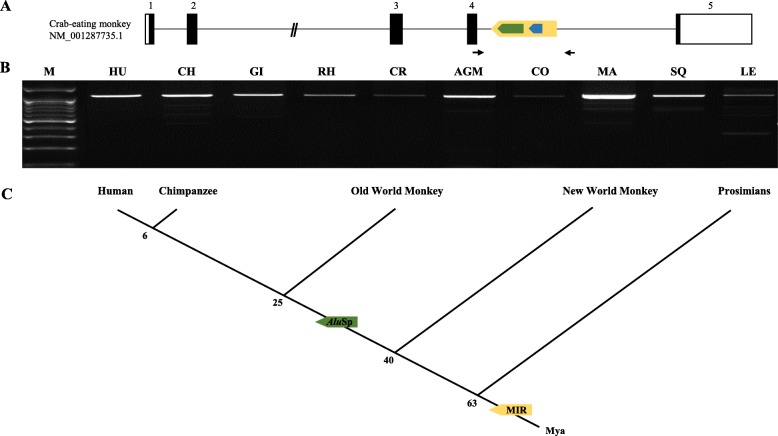
Integration of the MIR and *Alu*Sp elements in the *BLOC1S2* gene during primate evolution. **a** Gene structure of the crab-eating monkey *BLOC1S2* gene and primer location. **b** PCR amplification of the MIR and *Alu*Sp elements in several primates. M indicates the size marker, and the primate names are abbreviated as follows. HU: human; CH: chimpanzee; GI: gibbon; RH: rhesus monkey; CR: crab-eating monkey; AGM: African green monkey; CO: colobus monkey; MA: marmoset; SQ: squirrel monkey; and LE: ring-tailed lemur. **c** Schematic representation of the integration event of MIR and *Alu*Sp into the *BLOC1S2* gene during primate evolution. Green and yellow arrow boxes represent the *Alu*Sp and MIR elements, respectively. Mya: millions of years ago

To investigate the integration time of the target MIR and *Alu*Sp element, we experimentally analyzed genomic DNA samples from 10 primates, including hominoids (human, chimpanzee, and gibbon), Old World monkeys (rhesus monkey, crab-eating monkey, African green monkey, and colobus monkey), New World monkeys (marmoset and squirrel monkey), and prosimian (ring-tailed lemur). We performed genomic PCR amplification of these samples (Fig. 2a and b) and obtained their sequences using gene-cloning methods. The alignment of these sequences revealed that the MIR is located in an antisense-orientation on the 4th intron of the *BLOC1S2* gene in all tested primates and that the antisense-oriented *Alu*Sp is located in the same region, but only in the hominoids and Old World monkeys (Additional file 1: Figure S2). Notably, *Alu*Sp exists inside the MIR sequence. Unlike the hominoids and Old World monkeys, the New World monkeys and prosimians do not contain a 260 bp *Alu*Sp element, but possess a different *Alu*s at different loci within the target PCR products, which results in similar sizes of the 10 main PCR amplicons (Fig. 2b and Additional file 1: Figure S3). Taken together, these evolutionary analyses indicate that the MIR element was integrated into the genome of simians and prosimians prior to the divergence of their common ancestor, approximately more than 63 million years (myrs) ago. In addition, the *Alu*Sp element was integrated into the genome of hominoids and Old World monkeys after they split off from their common ancestors with New World monkeys somewhere between 25 and 40 myrs ago (Fig. 2c).

### Experimental validation of MIR_*Alu*-derived exonization events in the *BLOC1S2* gene

**Fig. 3:**
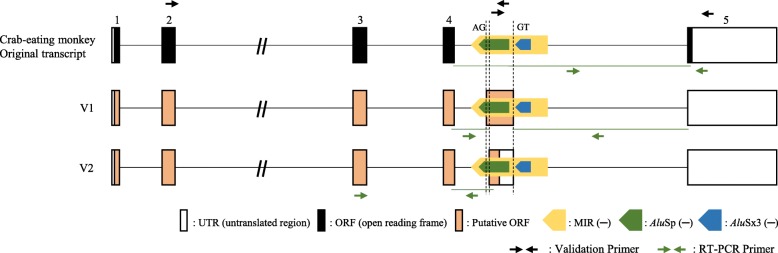
Structural analysis of *BLOC1S2* gene transcripts in the crab-eating monkey, rhesus monkey, and African green monkey through RT-PCR experiments and sequencing of the products. The two transcript variants (V1 and V2) attributed to MIR_*Alu*Sp-derived exonization events were identified in all species. Each arrow box with a different color indicates a different SINE element. Open and closed boxes represent the untranslated region of the exon and coding sequence, respectively. Yellow, green, and blue arrow boxes represent MIR, *Alu*Sp, and *Alu*Sx3, respectively. Black and green small arrows represent the validation primer and RT-PCR primers, respectively. This figure is a structural illustration and is not drawn to scale

Both ends of the newly detected partial exon were not correctly identified, making it necessary to perform RT-PCR experiments to validate both ends. Tissue cDNA samples from the crab-eating monkey were used, and two primer pairs were designed: i) Validation Primer 1 was used to find the 5′-end of the partial exon and 2) Validation Primer 2 was used to check the 3′-end of this partial exon (Additional file 1: Table S1). As a result, we clarified the sequence of the full length of the partial exon, finding that the 5′-end is spliced at the 3′-SS (AG) derived from the *Alu*Sp sequence on the 4th intron of the gene and that the 3′-end is spliced at the 5′-SS (GT) derived from the MIR sequence on the same intron (Fig. 3 and Additional file 1: Figure S2). We named this exon V1. In addition, to determine if the exon V1 exists in other species of primates, several more rounds of RT-PCR and sequencing were carried out with the available human, chimpanzee, gibbon, rhesus monkey, and African green monkey tissues (Additional file 1: Figure S4). The V1 transcript was detected in the rhesus monkey and African green monkey, but not in human, chimpanzee, and gibbon (Additional file 1: Figure S4A). For human, chimpanzee, and gibbon, numerous non-specific amplicons were detected (Additional file 1: Figure S4B); therefore, several of these amplicons of acceptable size were sequenced, but they were unrelated to V1. Furthermore, we found that a 506 bp amplicon that was exon V1 but with a different 3′-SS (AG) loci provided by the same *Alu*Sp in the cerebrum of the African green monkey (Additional file 1: Figure S4A and Fig. 3). We named it V2, and a new primer pair, Primer 3 (Additional file 1: Table S1), was applied to check the ends of V2. We confirmed that V2 had a different 3′-SS, and its expression was experimentally validated in the African green monkey, crab-eating monkey, and rhesus monkey, but not in human, chimpanzee, and gibbon (Additional file 1: Figure S5).

### Expression patterns of the MIR_*Alu*-derived exon in various primates

**Fig. 4:**
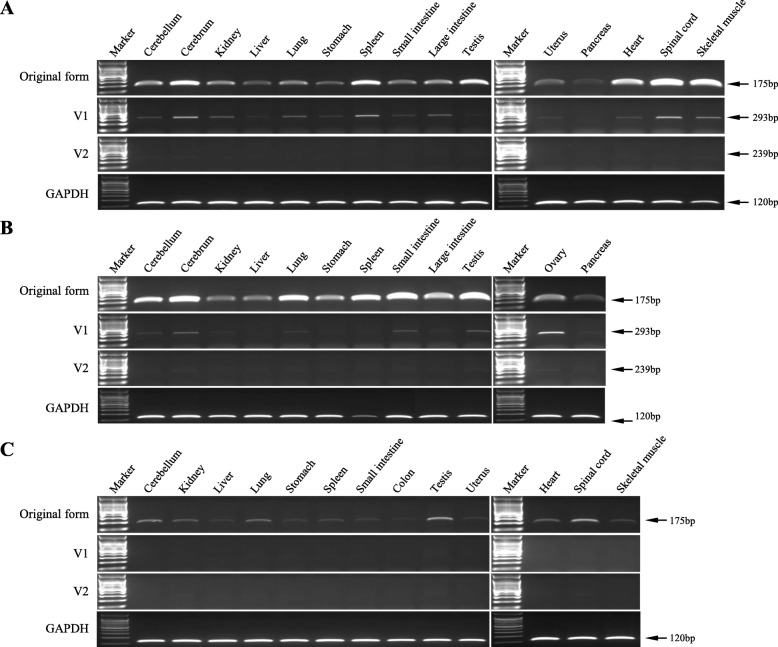
RT-PCR amplification for expression pattern analysis of the *BLOC1S2* gene in the crab-eating monkey (**a**), rhesus monkey (**b**), and human (**c**). Original transcript (175 bp), V1 (293 bp), and V2 (239 bp) were amplified using transcript-specific set of primers, Primers 1, 2, and 3, respectively. *GAPDH* (120 bp) indicates the positive control. In case of V2, see Additional file 1: Figure S6 to check band presence

To clarify the expression patterns of the original, V1, and V2 transcripts, we performed RT- PCR amplification of this region using various tissue cDNA samples from the crab-eating monkey, rhesus monkey, and human. The results revealed a ubiquitous expression pattern of the original transcript in all tissue samples from the crab-eating monkey, rhesus monkey, and human; however, the expression level was relatively low in the pancreas of the crab-eating monkey, the kidney, and liver of the rhesus monkey, and the human liver and colon (Fig. 4). V1 was expressed in all the tissue samples from the crab-eating monkey, with a low level of expression in the pancreas. However, very low or no V1 expression was found in the tissue samples from the rhesus monkey. In case of human tissues, V1 was not detected in any of the tissue samples analyzed (Fig. 4c). Thus, we designed and applied a new primer called Primer 5 (Additional file 1: Table S1) to detect V1 more precisely in case there were very low expression levels in human tissues, but no variants related to V1 were detected (data not shown). RT-PCR was used to detect V2 with the same tissue cDNA samples, and the results revealed that V2 is partly expressed in tissue samples from the crab-eating monkey and rhesus monkey (Fig. 4 and Additional file 1: Figure S6). Specifically, no V2 was expressed in the kidney, stomach, spleen, small intestine, pancreas, heart, and spinal cord of the crab-eating monkey and in the kidney, liver, stomach, and pancreas of the rhesus monkey. On the other hand, V2 was expressed in the cerebellum, cerebrum, lung, large intestine, and testis of the two species (Additional file 1: Figure S6). In human tissues, we were able to detect six faint amplicons that were of a similar size (277 bp) to the target band (239 bp) (Additional file 1: Figure S6), but we confirmed by cloning that these bands were not the target V2.

### Peptide sequence analysis of *BLOC1S2*

**Fig. 5:**
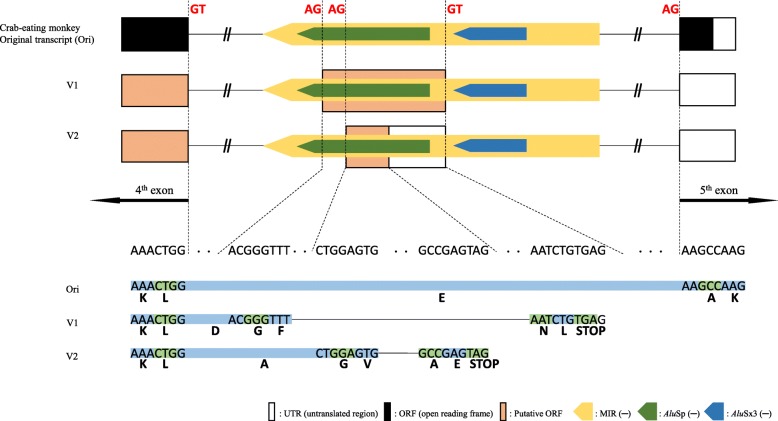
Transcript and peptide sequence analysis of splice site of V1 and V2 compared to the crab-eating monkey original transcript (NM_001287735.1). Bold red text and Bold black letter indicate nucleotide sequence of splice site and 1-letter abbreviation of amino acid, respectively. For the full amino acid sequences, see Additional file 1: Figure S7

The translated sequence analysis using the ORF finder program revealed that the original, V1, and V2 transcripts encode 142, 222, and 160 amino acids, respectively (Additional file 1: Figure S7). Additionally, domain search analysis using the Pfam database showed that a conserved domain named BLOC1_2 exists on the original transcript located from the 42nd amino acid to the 137th amino acid. For V1 and V2, however, the last five amino acids of this domain are replaced with a longer and different amino acid sequence (Additional file 1: Figure S7). A close look at these amino acid sequences being compared with the equivalent transcript sequences uncovered that these different amino acid sequences are derived from the inserted MIR_*Alu*Sp, providing premature stop codon (Fig. 5).

## Discussion

Non-human primates are very valuable animal models for biomedical research because of their biological, behavioral, physiological, and genetic similarities with humans [23, 24]. Among these primates, crab-eating monkeys, rhesus monkeys, and African green monkeys are the most representative and frequently used experimental animals in biomedical research [23]. These monkeys are Old World monkeys and diverged from the common ancestor of Old World monkeys and humans approximately 25–32 myrs ago [24].

TEs have moved around the genome over the past hundreds of million years and shaped the evolution of the human and non-human primate genomes [25, 26]. Previous studies have indicated that TE insertions are associated with more than 100 diseases to date because of their association with increasing genomic instability [27, 28]. Several disease-causing mechanisms have been identified such as insertional mutagenesis and aberrant splicing [25, 28]. On the other hand, TEs can be advantageous elements that increase genomic diversity, and have a tremendous impact on evolution at the DNA and RNA levels by transduction-mediated gene formation, gene retrotransposition, alternative splicing, alternative polyadenylation, and the actions of alternative promoters, enhancers, and silencer elements [11, 18, 25]. In the present study, for the first time, we identified and validated that MIR_*Alu*Sp combined sequences induced lineage-specific alternative splicing events and played an important role in diversifying *BLOC1S2* gene transcripts, especially in the crab-eating monkey, rhesus monkey, and African green monkey.

### Insertion mechanism of MIR and *Alu*Sp in *BLOC1S2*

The target exonization that was the subject of this study derives from combined sequences. Three SINE elements, two *Alus* and one MIR, are involved in this combination, and two of these elements, *Alu*Sp and MIR, are associated with the target exonization event. To investigate the collaborative mechanism involving these two TE insertion events, genomic DNA cloning and sequence analyses were conducted. The most plausible scenario for the integration event of the TEs based on thorough sequence analyses is as follows. The target MIR element was integrated into the 4th intron of the *BLOC1S2* gene in all analyzed primates prior to mammalian radiation [13], and then, *Alu*Sp was inserted into the endonuclease target motif located on the MIR of the catarrhine lineage before the divergence of hominoids and Old World monkeys, creating the combined MIR_*Alu*Sp sequence [20, 21].

The multiple alignment (Additional file 1: Figure S2) enabled us to analyze the insertion mechanism of the two elements. The main insertion mechanism that the SINE family usually adopts is called the target-primed reverse transcription (TPRT) [25, 29, 30]. This mechanism of insertion by *Alu* normally leaves the two following hallmarks in the genome: i) a target site duplication (TSD) and ii) a variable length poly-A tail [31]. The sequence analysis revealed that the target *Alu*Sp was inserted into the genome by the TPRT mechanism because it exhibited these two characteristics (Additional file 1: Figure S2). MIR has also been reported to have flanking repeats such as a TSD and an A/T-rich 3′-end, such as a poly-A-tail [20]. In the case of the A/T-rich 3′-end, it is still discernible. On the other hand, we were able to find three matched nucleotides directly in front and behind the MIR sequence that look like flanking repeats (Additional file 1: Figure S2). This could be the TSD according to a previous study that defined the length of a TSD as being between 2 and 20 bp [25]. Nevertheless, we cannot be sure that this is the TSD generated by MIR insertion because it seems to have become unrecognizable due to its 25–35% divergence from the consensus sequence [20].

### Alternative splicing events by combined TEs

Previous studies have shown that *Alu* and MIR elements were preferentially inserted in the antisense orientation and sense orientation, respectively, relative to the gene orientation. Moreover, 60 and 85% of the exonized sequences from MIR and *Alu*, respectively, are in the antisense orientation [16]. The genomic DNA sequence analysis performed in this study showed that the target MIR and *Alu*Sp elements were both in the antisense orientation and combined together and that two 3′-SS and one 5′-SS were derived from these elements, respectively (Figs. 3 and 5). Therefore, the combined MIR_*Alu*Sp elements in antisense-orientation can lead to AS events and exonization by providing potential splicing donor and acceptor sites in the intragenic regions. Thus, we conducted additional computational analyses to clarify the distribution of the combined MIR_*Alu*Sp elements at different loci in the genome of the crab-eating monkey, rhesus monkey, and human (Additional file 1: Table S2).

In the case of the MIR_*Alu*Sp combination of the crab-eating monkey, 209 cases were detected, and 60% (125cases) were in the same orientation. Additionally, we tried to calculate the number of MIR_*Alu*Sp combinations that were intragenic although the gene data files we used only contain NM_ and NR_genes that have been recently updated. As a result, 4 out of 125 cases, including the analyzed MIR_*Alu*Sp combination in the *BLOC1S2* gene, were both intragenic and intronic. The computing analyses of the rhesus monkey and human showed similar ratios, but different counts, because of the different number of registered genes for different species (Additional file 1: Table S2). This simple analysis revealed that combined MIR_*Alu*Sp sequences are distributed inside the genes in the crab-eating monkey, rhesus monkey, and human genomes. Further experiments to determine the number of transcripts created by MIR_*Alu*Sp sequences will reveal how much influence this specific combined sequence has on the genome. In addition, broad studies on various types of combinations coupled with their exonization may allow the identification of more TE-combined sequences that contribute to AS events and aid in the research on the preference of splice sites.

### Molecular mechanism of the MIR_*Alu*Sp sequence resulting in alternative splicing

Splicing is the process by which introns are removed from pre-mRNA transcripts, and three essential cis-acting elements, i) a 5′-SS, ii) 3′-SS, and iii) branch point (BP) are required for this intron removal process [32]. The process accompanies two transesterification reactions: the first reaction is involved in the attack of the BP adenosine on the 5′ splice donor to cut the intron from upstream exon, and the second reaction is involved in the attack of this detached end on the 3′ splice acceptor, releasing the intron in the form of a lariat [33]. Therefore, the BP recognized by the ribonucleoprotein U2 snRNA plays a crucial role in the initiation of splicing [34].

**Fig. 6:**
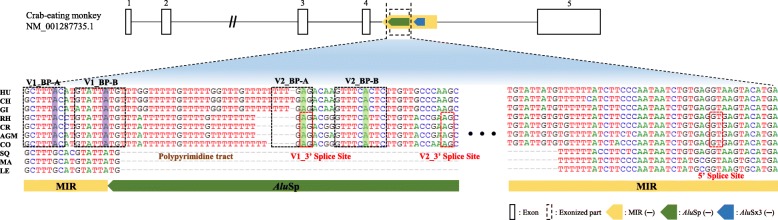
Branch point (BP) analysis of MIR_*Alu*Sp-derived V1 and V2 transcripts using the BPP and svm-BPfinder programs. The purple box is the predicted BP of the V1 transcript, and the green box is the predicted BP of the V2 transcript. The red dashed box represents 3′ splice sites and 5′ splice sites. Open and closed boxes represent the untranslated region of the exon and coding sequence, respectively. Yellow, green, and blue arrow boxes represent MIR, *Alu*Sp, and *Alu*Sx3, respectively. This figure is a structural illustration and is not drawn to scale

To analyze the 3′-SS of the newly identified target exon, we focused on the removal process of the upstream 4th intron. Computational analysis was performed to predict possible BP positions for this intron. For the V1 transcript, 5′-GCTTTAC-3′ (V1_BP-A) and 5′- GTATTATGT-3′ (V1_BP-B) were detected as the BP sequence by two different BP prediction tools, BPP and svm-BPfinder, respectively, and the last adenine of each BP sequence is expected to be the BP (Fig. 6). In the case of the V2 transcript, 5′-TTTTGAG-3′ (V2_BP-A) and 5′-GTTTCATTC-3′ (V2_BP-B) were identified as possible BP sequences, and the adenine of each BP sequence is considered as the BP (Fig. 6). A previous study reported that the human BP is usually found 21–25 nucleotides (nts) upstream of the 3′-SS or near this region [34–37]. The predicted BP position in this study was also detected near this region (Fig. 6). Interestingly, the upstream BP positions of the target exon in the hominoids and Old World monkeys are located in the same region, although the target exon is only expressed in the Old World monkeys. This may be because the predicted BP sequence of hominoids is not recognized by the U2 snRNP, inhibiting the creation of a downstream target exon.

A previous study revealed that BP selection is highly associated with the distance to the 3′-SS and BP binding stability [36]. First, a BP located farther from the 3′-SS than at the canonical BP position is less likely to be selected, resulting in the skipping of the downstream exon. Corvelo et al. showed that approximately 43% of the exons in which the BP was located more than 100 nts upstream of the 3′-SS were skipped, while only 28.6% of exons in which the BP was located less than 50 nts were skipped [36]. Second, the binding stability of the BP sequence to the binding sequence of the U2 snRNA could be another reason [36]. Recently, the binding energy of this base pairing was calculated with a computing program, which represents binding stability [36]. For example, a C to T mutation near the BP position of the *Fech1* gene intron that decreased the U2 binding energy was reported as the cause of the creation of a new competing BP [38]. Likewise, in the case of the V2 transcript in this study, the BP binding energy in hominoids may be less than that in Old World monkeys. This is because the one nucleotide difference in the V2_BP-B between the hominoids (C) and Old World monkeys (T) could decrease its stability, hindering the predicted BP from being selected. In the case of the V1 transcript, the binding stability of a different protein, U2AF (U2 auxiliary factor), may be associated with this selection of the predicted BP. U2AF65, a 65-kDa subunit of U2AF, usually binds to the polypyrimidine tract (Py tract) right behind the BP sequence and is involved in the recruitment of U2 snRNP [39, 40]. In addition, a previous study revealed that the binding affinity of U2AF65 differs depending on the sequence of the Py tract [41]. Therefore, the different Py tract sequences between hominoids and Old World monkeys in this study could lower the U2AF65 binding energy, thereby interrupting the selection of the BP.

### Diverse peptide sequence derived from exonized MIR_*Alu*Sp

Our peptide sequence analysis of the target transcript using ORF finder program and Pfam database identified 2 more possible peptide sequences different from the one that is originated from the reference transcript (NM_001287735.1). It turns out, from the analysis, that these differences are generated by MIR_*Alu*Sp exonization and especially centered on C-terminal of these peptide sequences (Fig. 5 and Additional file 1: Figure S7). Previous studies have indicated that different C- terminal lengths of alternatively spliced isoform tend to result in different protein functions [42]. In addition, many predicted alternative transcripts might not be translated into proteins [43]. Thus, further studies are needed to validate whether the two *BLOC1S2* transcript variants are functional. Although a functional analysis was not performed in the present study, the specific integration and combination of the MIR and *Alu*Sp elements was shown to lead to lineage-specific AS events in the Old World monkeys. Therefore, in this study, we revealed a novel mechanism for exonization events involving collaboration of two different TEs, specifically in a lineage-specific manner.

## Conclusion

Overall, the combined sequence of the two MIR and *Alu*Sp elements in antisense orientation in the *BLOC1S2* gene favors Old World monkey-specific alternative splicing. In this molecular landscape, the MIR of the Old World monkey is evolutionally designed to provide a premature BP sequence. This BP sequence is also conducive to the determination of the 3′-SS on the *Alu*Sp sequence, contributing to the new exon generation inside the 4th intron. The subsequent intron removal process that starts at the 5′-SS on the MIR ends this newly detected target exon. Therefore, the merging of the two MIR and *Alu*Sp sequences in antisense orientation produced novel Old World monkey-specific transcript variants by adding a new exon to the *BLOC1S2* gene during primate evolution.

## Methods

### Total RNA and genomic DNA samples

Total RNA from human tissue samples (*Homo sapiens;* whole brain, kidney, liver, lung, stomach, spleen, small intestine, colon, testis, uterus, heart, spinal cord, and skeletal muscle) and rhesus monkey tissue samples (*Macaca mulatta;* cerebellum, cerebrum, kidney, liver, lung, stomach, spleen, small intestine, large intestine, testis, ovary, and pancreas) were purchased from Clontech Laboratories, Inc. Tissues from an adult female crab-eating monkey (*Macaca fascicularis;* cerebellum, cerebrum, kidney, liver, lung, stomach, spleen, small intestine, large intestine, testis, uterus, pancreas, heart, spinal cord, and skeletal muscle) that originated from Vietnam was imported from China under a Convention on International Trade in Endangered Species of Wild Fauna and Flora (CITES) permit. The crab-eating monkey was provided by the National Primate Research Center (NPRC) of the Republic of Korea. We used a standard protocol to isolate genomic DNA from heparinized blood samples from the following species: (1) hominoids: HU, humans (*Homo sapiens*); CH, chimpanzees (*Pan troglodytes*); and GI, gibbon (*Symphalangus syndactylus*); (2) Old World monkeys: RH, rhesus monkeys (*Macaca mulatta*), CR, crab-eating monkeys (*Macaca fascicularis*), AGM, African green monkeys (*Chlorocebus aethiops*), and CO, colobus monkeys (*Procolobus badius*); (3) New World monkeys: MAR, marmosets (*Callithrix jacchus*) and SQ, squirrel monkeys (*Saimiri sciureus*); and (4) prosimians: LE, ring-tailed lemurs (*Lemur catta*).

### RT-PCR and PCR amplification

*BLOC1S2* transcripts were analyzed by RT-PCR amplification using the GoScript Reverse Transcriptase (RT) System (Promega) with an annealing temperature of 42 °C. We performed PCR amplification of pure mRNA samples without reverse transcription to demonstrate that the mRNA samples did not contain genomic DNA (data not shown). The expression levels of the *BLOC1S2* gene of humans, rhesus monkeys, and crab-eating monkeys were tested by RT-PCR experiments using specific primer pairs (Additional file 1: Table S1). RT-PCR experiments were carried out for 30 cycles of 94 °C for 30 s, 59 °C for 30 s, and 72 °C for 30 s. We additionally performed 35 cycles of RT-PCR experiments for V2 transcript due to its dimly visible bands with 30 cycles. Genomic DNA from the different primates was divided into two groups and separately amplified with two different primer pairs, one for human, chimpanzee, and gibbon and another for rhesus monkey, crab-eating monkey, African green monkey, marmoset, squirrel monkey, and ring-tailed lemur. The genomic PCR conditions for the former group were 35 cycles of 94 °C for 30 s, 58 °C for 30 s, and 72 °C for 70 s, and those for the latter group were 34 cycles of 94 °C for 30 s, 58 °C and 62 °C for 30 s, and 72 °C for 60 s.

### Molecular cloning and sequencing procedures

PCR products were separated on a 1.2% agarose gel, purified with the Gel SV Extraction kit (GeneAll), and cloned into the TA cloning vector (RBC Bioscience). The cloned DNA was isolated using the Hybrid-Q™ Plasmid mini-prep kit (GeneAll). Sequencing of primate DNA samples and alternative transcripts was performed by a commercial sequencing company (Macrogen Inc).

### Branch point analysis

Branch point (BP) analysis was performed using two different programs, svm-BPfinder [36], a widely used program, and BPP, the most recently developed program [34]. These operate on a website and the Linux operating system, respectively. The input sequences ranged from the GT dinucleotide, at the 5′-end of the 4th intron of the *BLOC1S2* gene, to the AG dinucleotide, a 3′-SS of the target exonized sequence, and genomic PCR sequences of human, chimpanzee, gibbon, crab-eating monkey, rhesus monkey, and African green monkey were used for the analysis. In svm-BPfinder, although many possible BP sequences were predicted based on the distance from the 3′-SS and scoring information, the most reliable BP sequence that was connected to the target 3′-SS was manually selected according to the instructions of the program. On the other hand, BPP gave only one reliable BP sequence with the programed scoring information.

### Combined sequence analysis

TinT, screening program for nested transposition was introduced in the paper of Churakov et al. [44]. In the program, input file must be RepeatMasker outfile but up-to-date outfile in RepeatMasker website was generated in 2010. Even the outfile used as an input file for the TinT program in the paper of Churakov et al. was generated in 2006. In this study, therefore, we decided to obtain the desired information using our own algorithm on Linux with recently (2016) generated files instead of using TinT program. We calculated the number of TE combination phenomena using Awk, and Python languages in the Linux operating system (Ubuntu 17.04) with the recently annotated data files from the UCSC database. We downloaded repeat-masking files (rmsk.txt.gz) and reference gene files (refGene.txt.gz) for crab-eating monkey, rhesus monkey, and human from ftp://hgdownload.soe.ucsc.edu. We simply parsed the repeat-masking file to count the total number of TEs, SINEs, *Alu*Sp elements, and MIR elements and built a Python program algorithm to extract all the combinations in which the TEs are together. Finally, we determined the total number of MIR_*Alu*Sp combinations and compared this information with a reference gene file to check how many intragenic MIR_*Alu*Sp combinations there were. We applied the same algorithm to all three species.

### Peptide sequence analysis

Peptide sequence analysis was performed with ORF finder program and Pfam database. ORF finder program in NCBI website (www.ncbi.nlm.nih.gov/orffinder) is a graphical analysis tool searching for open reading frames (ORFs). To search for possible ORFs and protein sequences of orignal transcript, V1 and V2 respectively, we used each predicted nucleotide sequence as a query sequence. Of the resulting ORFs, we selected one that starts first which could most likely be translated. Afterwards, we do the domain search analysis in the Pfam database website (pfam.xfam.org). In the Pfam website, each protein sequence of the selected ORF is used as a query sequence.

## Supplementary information

**Additional file 1: Table S1.** Primer list for genomic PCR and RT-PCR. **Figure S1.** Alignment of reported *BLOC1S2* m-RNA sequences for crab-eating monkey, rhesus monkey and human. **Figure S2.** Multiple sequence alignment of the integrated MIR and *Alu*Sp region in the *BLOC1S2* gene. **Figure S3.** Structural analysis of gDNA PCR products of 10 primates. **Figure S4.** RT-PCR amplification for validation of the MIR_*Alu*Sp-derived V1 transcript. The 5′- and 3′-ends of MIR_*Alu*Sp-derived exon were validated using Validation Primers 1 and 2. A) 531 bp product and 389 bp product of the V1 transcript were detected in the crab-eating monkey, rhesus monkey, and African green monkey. For the African green monkey, a 506 bp product of a different 5′-end transcript (V2) was detected. B) The 531 bp product and 389 bp product of the V1 transcript were not detected in the human, chimpanzee, and gibbon samples. **Figure S5.** RT-PCR amplification for validation of the V2 transcript using Primer 3. A 239 bp product of the V2 transcript was detected in the crab-eating monkey, rhesus monkey, and African green monkey and was not detected in human, chimpanzee, and gibbon samples. **Figure S6.** RT-PCR amplification for expression pattern of V2 transcript (239 bp) in the crab-eating monkey (A), rhesus monkey (B), and human (C). The experiments were performed with 35 cycles of 94 °C for 30 s, 59 °C for 30 s, and 72 °C for 30 s because 30 cycles of V2 PCR amplification in Fig. 4 doesn’t show clear target bands. **Figure S7.** Multiple alignment of the amino acid sequences of the reference transcript, V1, and V2 transcripts of *BLOC1S2*. Lysine (K), Leucine (L) in green rectangular part are equivalent to the last part of the 4th exon nucleotide sequences of *BLOC1S2* transcript and see Fig. 5 for more details. **Table S2.** Calculation of MIR_AluSp combination throughout the genome.

## Data Availability

All data generated or analysed during this study are included in this published article [and its supplementary information files].

## Abbreviations

3′-SS: 3′-splicing site
5′-SS: 5′-splicing site
AS: Alternative splicing
BP: Branch point
CDS: Coding sequence
HERV: Human endogenous retrovirus
LINE: Long interspersed nuclear elements
NHP: Non-human primate
NWM: New World monkey
OWM: Old World monkey
SINE: Short interspersed nuclear elements
TEs: Transposable elements
TPRT: Target-primed reverse transcription
TSD: Target site duplication
UTR: Untranslated region

## References

[1] Zhou W, He Q, Zhang C, He X, Cui Z, Liu F, Li W. BLOS2 negatively regulates Notch signaling during neural and hematopoietic stem and progenitor cell development. Elife. 2016;5. 10.7554/eLife.18108. 10.7554/eLife.18108 PMC5094856 27719760

[2] Gdynia G, Lehmann-Koch J, Sieber S, Tagscherer KE, Fassl A, Zentgraf H, Matsuzawa S, Reed JC, Roth W (2008). BLOC1S2 interacts with the HIPPI protein and sensitizes NCH89 glioblastoma cells to apoptosis. Apoptosis.

[3] Sun J, Nie J, Hao B, Li L, Xing G, Wang Z, Zhou Y, Sun Q, Li G, Zhang L (2008). Ceap/BLOS2 interacts with BRD7 and selectively inhibits its transcription-suppressing effect on cellular proliferation-associated genes. Cell Signal.

[4] Sammeth M, Foissac S, Guigo R (2008). A general definition and nomenclature for alternative splicing events. PLoS Comput Biol.

[5] Iniguez LP, Hernandez G (2017). The evolutionary relationship between alternative splicing and gene duplication. Front Genet.

[6] Gallego-Paez LM, Bordone MC, Leote AC, Saraiva-Agostinho N, Ascensao-Ferreira M, Barbosa-Morais NL (2017). Alternative splicing: the pledge, the turn, and the prestige : the key role of alternative splicing in human biological systems. Hum Genet.

[7] Ast G (2004). How did alternative splicing evolve?. Nat Rev Genet.

[8] Kim E, Goren A, Ast G (2008). Alternative splicing: current perspectives. Bioessays.

[9] Kim YH, Choe SH, Song BS, Park SJ, Kim MJ, Park YH, Yoon SB, Lee Y, Jin YB, Sim BW (2016). Macaca specific exon creation event generates a novel ZKSCAN5 transcript. Gene.

[10] Lev-Maor G, Sorek R, Shomron N, Ast G (2003). The birth of an alternatively spliced exon: 3′ splice-site selection in Alu exons. Science.

[11] Park SJ, Kim YH, Lee SR, Choe SH, Kim MJ, Kim SU, Kim JS, Sim BW, Song BS, Jeong KJ (2015). Gain of a new exon by a lineage-specific Alu element-integration event in the BCS1L gene during primate evolution. Mol Cells.

[12] Keren H, Lev-Maor G, Ast G (2010). Alternative splicing and evolution: diversification, exon definition and function. Nat Rev Genet.

[13] Jjingo D, Conley AB, Wang J, Marino-Ramirez L, Lunyak VV, Jordan IK (2014). Mammalian-wide interspersed repeat (MIR)-derived enhancers and the regulation of human gene expression. Mob DNA.

[14] Bannert N, Kurth R (2004). Retroelements and the human genome: new perspectives on an old relation. Proc Natl Acad Sci U S A.

[15] Schmitz J, Brosius J (2011). Exonization of transposed elements: a challenge and opportunity for evolution. Biochimie.

[16] Krull M, Petrusma M, Makalowski W, Brosius J, Schmitz J (2007). Functional persistence of exonized mammalian-wide interspersed repeat elements (MIRs). Genome Res.

[17] Gal-Mark N, Schwartz S, Ast G (2008). Alternative splicing of Alu exons--two arms are better than one. Nucleic Acids Res.

[18] Lee JR, Park SJ, Kim YH, Choe SH, Cho HM, Lee SR, Kim SU, Kim JS, Sim BW, Song BS (2017). Alu-derived alternative splicing events specific to Macaca lineages in CTSF gene. Mol Cells.

[19] Sorek R, Ast G, Graur D (2002). Alu-containing exons are alternatively spliced. Genome Res.

[20] Smit AF, Riggs AD (1995). MIRs are classic, tRNA-derived SINEs that amplified before the mammalian radiation. Nucleic Acids Res.

[21] Carnevali D, Conti A, Pellegrini M, Dieci G (2017). Whole-genome expression analysis of mammalian-wide interspersed repeat elements in human cell lines. DNA Res.

[22] Huh JW, Kim YH, Park SJ, Kim DS, Lee SR, Kim KM, Jeong KJ, Kim JS, Song BS, Sim BW (2012). Large-scale transcriptome sequencing and gene analyses in the crab-eating macaque (Macaca fascicularis) for biomedical research. BMC Genomics.

[23] Lee JR, Ryu DS, Park SJ, Choe SH, Cho HM, Lee SR, Kim SU, Kim YH, Huh JW (2018). Successful application of human-based methyl capture sequencing for methylome analysis in non-human primate models. BMC Genomics.

[24] Kim YH, Park SJ, Choe SH, Lee JR, Cho HM, Kim SU, Kim JS, Sim BW, Song BS, Lee Y (2017). Identification and characterization of the tyrosinase gene (TYR) and its transcript variants (TYR_1 and TYR_2) in the crab-eating macaque (Macaca fascicularis). Gene.

[25] Cordaux R, Batzer MA (2009). The impact of retrotransposons on human genome evolution. Nat Rev Genet.

[26] Lee JR, Kim YH, Park SJ, Choe SH, Cho HM, Lee SR, Kim SU, Kim JS, Sim BW, Song BS (2016). Identification of alternative variants and insertion of the novel polymorphic AluYl17 in TSEN54 gene during primate evolution. Int J Genomics.

[27] Platt RN, Vandewege MW, Ray DA (2018). Mammalian transposable elements and their impacts on genome evolution. Chromosom Res.

[28] Hancks DC, Kazazian HH (2016). Roles for retrotransposon insertions in human disease. Mob DNA.

[29] Kryatova MS, Steranka JP, Burns KH, Payer LM (2017). Insertion and deletion polymorphisms of the ancient AluS family in the human genome. Mob DNA.

[30] Dewannieux M, Esnault C, Heidmann T (2003). LINE-mediated retrotransposition of marked Alu sequences. Nat Genet.

[31] Ade C, Roy-Engel AM, Deininger PL (2013). Alu elements: an intrinsic source of human genome instability. Curr Opin Virol.

[32] Tan J, Ho JX, Zhong Z, Luo S, Chen G, Roca X (2016). Noncanonical registers and base pairs in human 5′ splice-site selection. Nucleic Acids Res.

[33] Bitton DA, Rallis C, Jeffares DC, Smith GC, Chen YY, Codlin S, Marguerat S, Bahler J (2014). LaSSO, a strategy for genome-wide mapping of intronic lariats and branch points using RNA-seq. Genome Res.

[34] Zhang Q, Fan X, Wang Y, Sun M, Shao J, Guo D (2017). BPP: a sequence-based algorithm for branch point prediction. Bioinformatics.

[35] Mackereth CD, Madl T, Bonnal S, Simon B, Zanier K, Gasch A, Rybin V, Valcarcel J, Sattler M (2011). Multi-domain conformational selection underlies pre-mRNA splicing regulation by U2AF. Nature.

[36] Corvelo A, Hallegger M, Smith CW, Eyras E (2010). Genome-wide association between branch point properties and alternative splicing. PLoS Comput Biol.

[37] Mercer TR, Clark MB, Andersen SB, Brunck ME, Haerty W, Crawford J, Taft RJ, Nielsen LK, Dinger ME, Mattick JS (2015). Genome-wide discovery of human splicing branchpoints. Genome Res.

[38] Signal B, Gloss BS, Dinger ME, Mercer TR (2018). Machine learning annotation of human branchpoints. Bioinformatics.

[39] Shao C, Yang B, Wu T, Huang J, Tang P, Zhou Y, Zhou J, Qiu J, Jiang L, Li H (2014). Mechanisms for U2AF to define 3′ splice sites and regulate alternative splicing in the human genome. Nat Struct Mol Biol.

[40] Chen L, Weinmeister R, Kralovicova J, Eperon LP, Vorechovsky I, Hudson AJ, Eperon IC (2017). Stoichiometries of U2AF35, U2AF65 and U2 snRNP reveal new early spliceosome assembly pathways. Nucleic Acids Res.

[41] Cho S, Moon H, Loh TJ, Jang HN, Liu Y, Zhou J, Ohn T, Zheng X, Shen H (2015). Splicing inhibition of U2AF65 leads to alternative exon skipping. Proc Natl Acad Sci U S A.

[42] Hilgard P, Huang T, Wolkoff AW, Stockert RJ (2002). Translated Alu sequence determines nuclear localization of a novel catalytic subunit of casein kinase 2. Am J Physiol Cell Physiol.

[43] Tress ML, Abascal F, Valencia A (2017). Alternative splicing may not be the key to proteome complexity. Trends Biochem Sci.

[44] Churakov G, Grundmann N, Kuritzin A, Brosius J, Makalowski W, Schmitz J (2010). A novel web-based TinT application and the chronology of the primate Alu retroposon activity. BMC Evol Biol.

