# Supplementary material for: Cooperative evolution of two different TEs results in lineage-specific novel transcripts in the BLOC1S2 gene
Source: BMC Evol Biol. 2019 Oct 30;19:196. doi: 10.1186/s12862-019-1530-0 (PMC6822395; doi:10.1186/s12862-019-1530-0)
Supplement: Supplementary file 1 — Additional file 1: Table S1. Primer list for genomic PCR and RT-PCR. Figure S1. Alignment of reported BLOC1S2 m-RNA sequences for crab-eating monkey, rhesus monkey and human. Figure S2. Multiple sequence alignment of the integrated MIR and AluSp region in the BLOC1S2 gene. Figure S3. Structural analysis of gDNA PCR products of 10 primates. Figure S4. RT-PCR amplification for validation of the MIR_AluSp-derived V1 transcript. The 5′- and 3′-ends of MIR_AluSp-derived exon were validated using Validation Primers 1 and 2. A) 531 bp product and 389 bp product of the V1 transcript were detected in the crab-eating monkey, rhesus monkey, and African green monkey. For the African green monkey, a 506 bp product of a different 5′-end transcript (V2) was detected. B) The 531 bp product and 389 bp product of the V1 transcript were not detected in the human, chimpanzee, and gibbon samples. Figure S5. RT-PCR amplification for validation of the V2 transcript using Primer 3. A 239 bp product of the V2 transcript was detected in the crab-eating monkey, rhesus monkey, and African green monkey and was not detected in human, chimpanzee, and gibbon samples. Figure S6. RT-PCR amplification for expression pattern of V2 transcript (239 bp) in the crab-eating monkey (A), rhesus monkey (B), and human (C). The experiments were performed with 35 cycles of 94 °C for 30 s, 59 °C for 30 s, and 72 °C for 30 s because 30 cycles of V2 PCR amplification in Fig. 4 doesn’t show clear target bands. Figure S7. Multiple alignment of the amino acid sequences of the reference transcript, V1, and V2 transcripts of BLOC1S2. Lysine (K), Leucine (L) in green rectangular part are equivalent to the last part of the 4th exon nucleotide sequences of BLOC1S2 transcript and see Fig. 5 for more details. Table S2. Calculation of MIR_AluSp combination throughout the genome. [file 12862_2019_1530_MOESM1_ESM.pdf]

Table 1. Primer list for genomic PCR and RT-PCR.

PRIMER		SEQUENCE	Amplicon Size (bp)	NOTE	
Validation Primer 1	Forward	5’- CTG AAG CTG ACA TCA CTG AGC - 3’	531	Validation of 5' end exon	RT-PCR
	Reverse	5’ - GTC GAG GTA GGC AGA TCA CC - 3’			
Validation Primer 2	Forward	5’ – AAG CGA TTC TCC TGC CTC AG - 3’	389	Validation of 3' end exon	
	Reverse	5’ – AGC CAC AGT CCT CCA GTT TA - 3’			
Primer 1	Forward	5’ – ACT GGA AGC CAA GTA CAA GA - 3’	175	original form	
	Reverse	5’ – ACG TTG AGA TGT TCC TGG GA - 3’			
Primer 2	Forward	5’ – AAA ACT GGA CGG GTT TCA TTC- 3’	293	Alternative splicing form (V1)	
	Reverse	5’ – TGT ACT TGG CTT CTC ACA GA - 3’			
Primer 3	Forward	5’ – CCA CCA GTG AAG ACT ATA AGC TC - 3’	239	Check different loci of 3' spliced site (V2)	
	Reverse	5’ – CCA TTG CAC TCC AGC CAG T - 3’			
Primer 4	Forward	5’ – CCA CCA GTG AAG ACT ATA AGC T - 3’	240	Check different loci of 3' spliced site (V1)	
	Reverse	5’ – AAG AAT GAA ACC CGT CCA GT - 3’			
Primer 5	Forward	5’ – ACT CAC CAG CTT GAA GTA TCT TG - 3’	530	Human-specific primer to check AS event in Human	
	Reverse	5’ – GGC AGG AGA ATC GCT TGA AC - 3’			
Genomic Primer 1	Forward	5’ – ACC ATG TAT GTA AGA GCC AGA G - 3’	-	Human sequence -based (Hu, Ch, Go)	gDNA-PCR
	Reverse	5’ – GCT GGA CTG CAG CCT TAT CT - 3’			
Genomic Primer 2	Forward	5’ – ACC ATG TGT ATA AGA GCC AGA G - 3’	-	Marmoset sequence - based (Rh, Cr, Agm, Co, Ma, Sq, Le)	
	Reverse	5’ – GCT GGA CTG CAG CCT TAT CT - 3’			

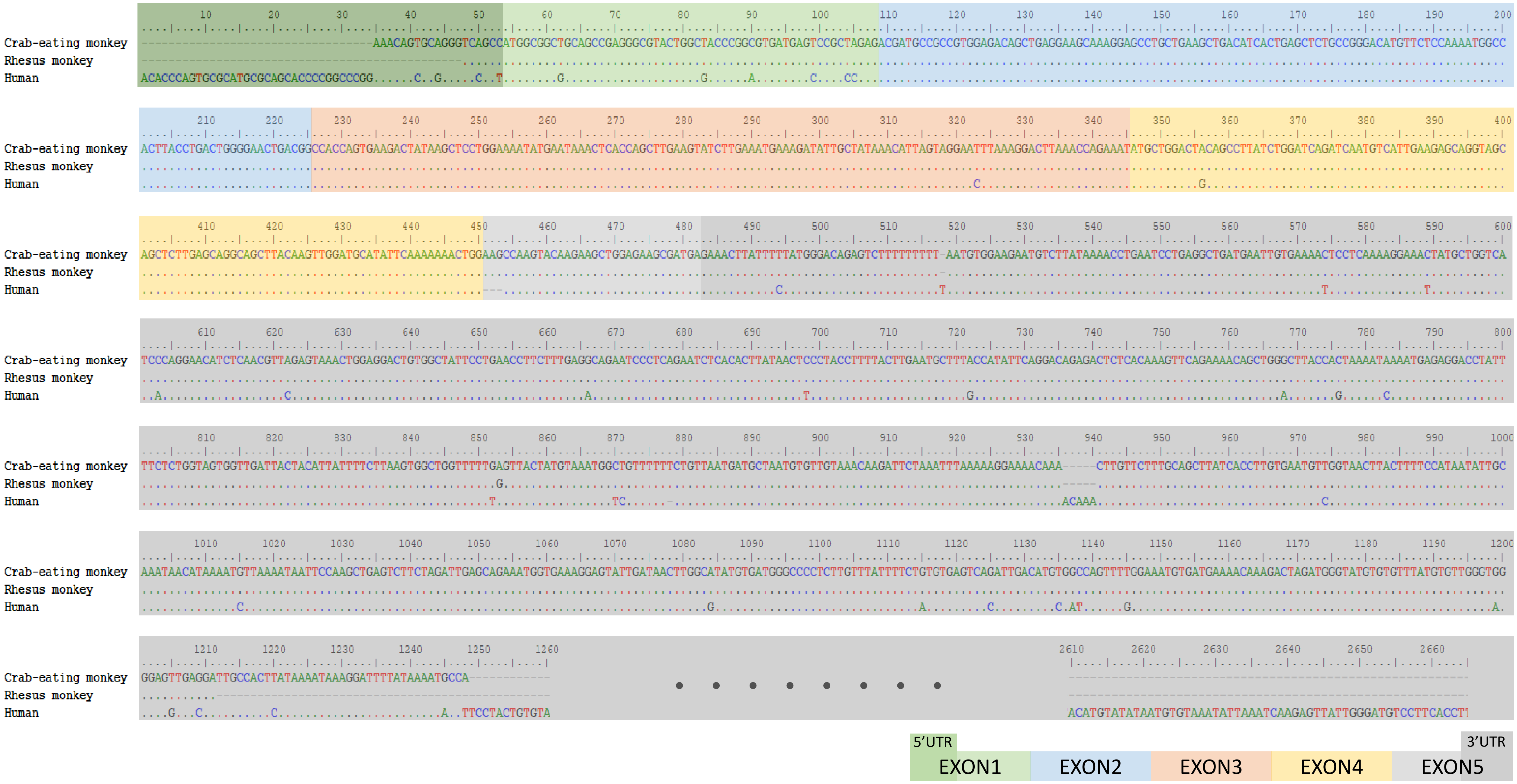

Figure S1. Alignment of reported *BLOC1S2* m-RNA sequences for crab-eating monkey, rhesus monkey and human.

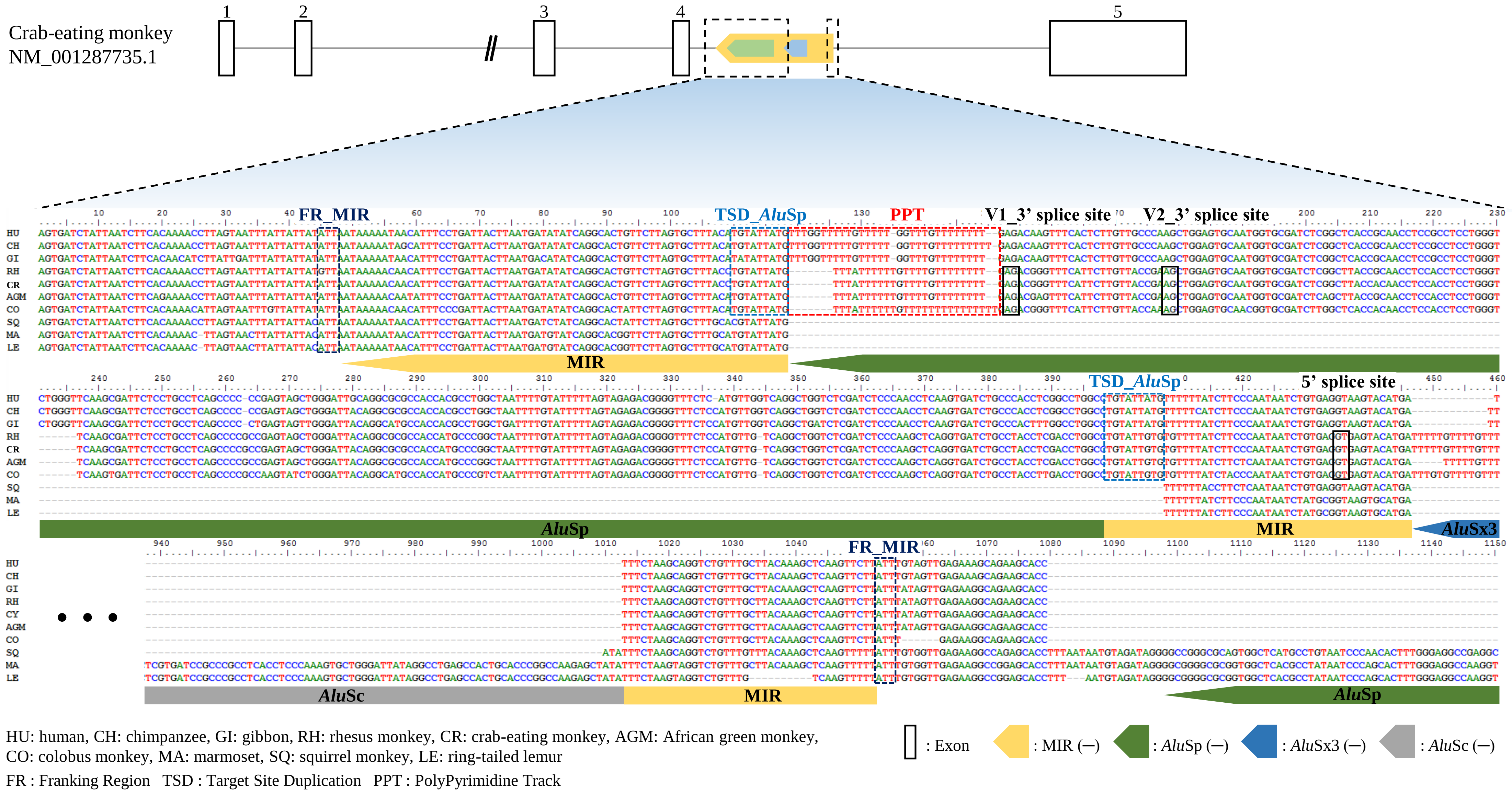

Figure S2. Multiple sequence alignment of the integrated MIR and *Alu*Sp region in the *BLOC1S2* gene.

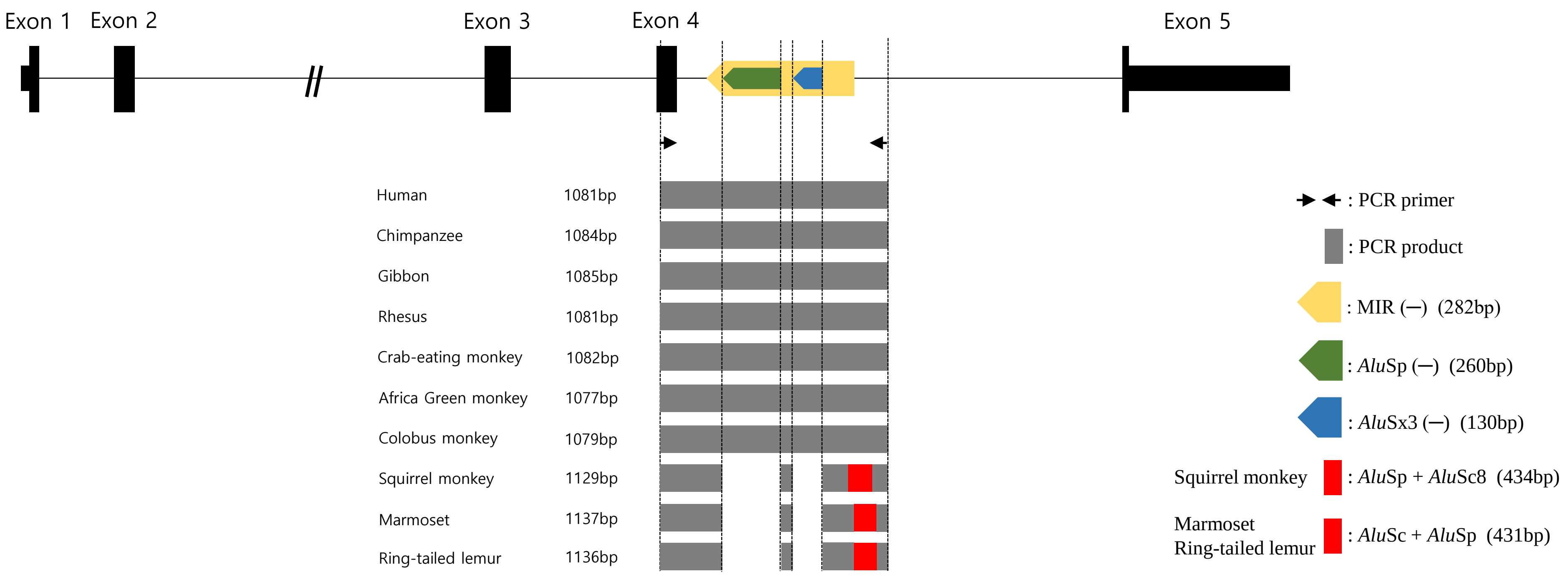

Figure S3. Structural analysis of gDNA PCR products of 10 primates.

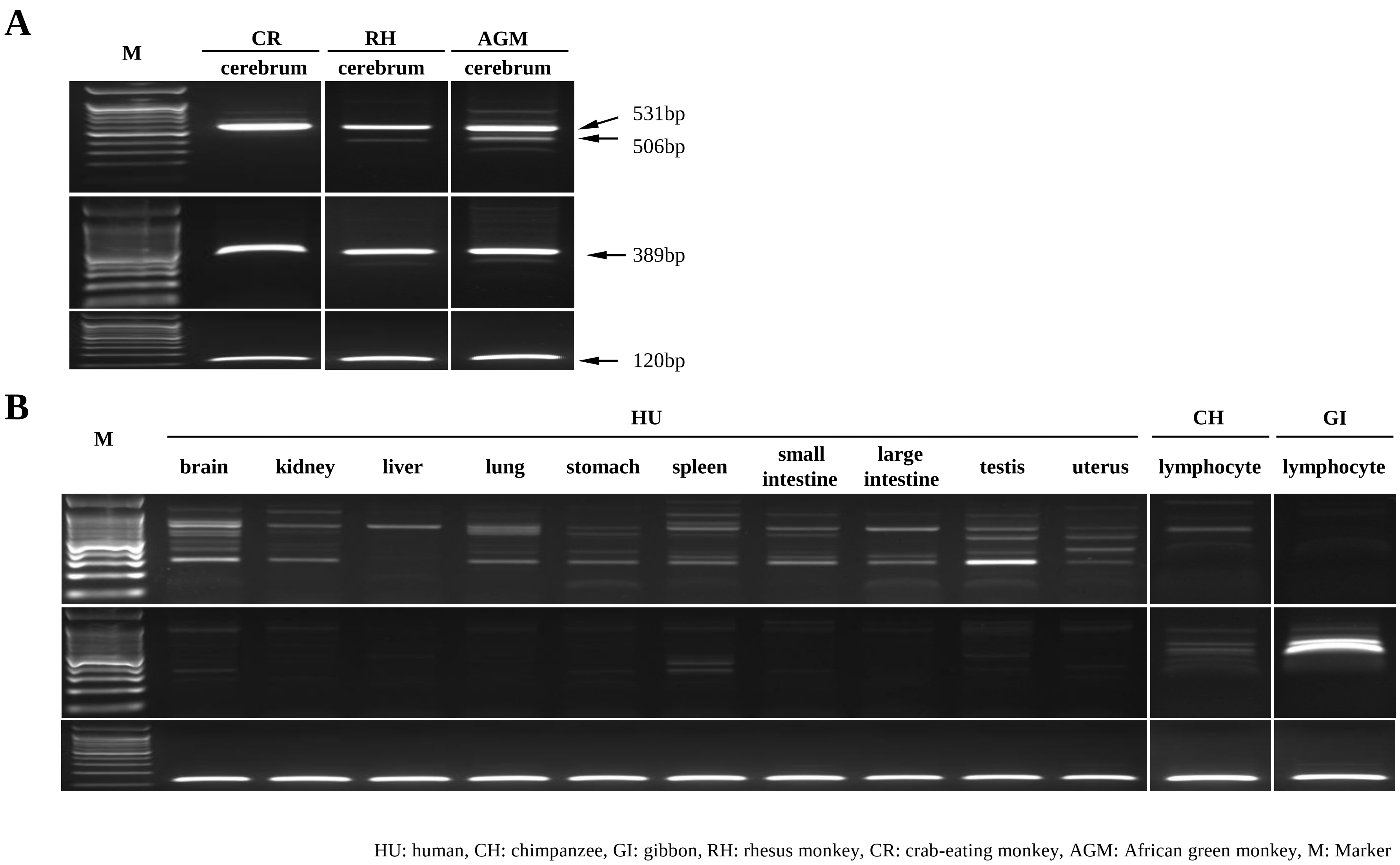

Figure S4. RT-PCR amplification for validation of the MIR_*Alu*Sp-derived V1 transcript. The 5′- and 3′-ends of MIR_*Alu*Sp-derived exon were validated using Validation Primers 1 and 2. A) 531 bp product and 389 bp product of the V1 transcript were detected in the crab-eating monkey, rhesus monkey, and African green monkey. For the African green monkey, a 506 bp product of a different 5′-end transcript (V2) was detected. B) The 531 bp product and 389 bp product of the V1 transcript were not detected in the human, chimpanzee, and gibbon samples.

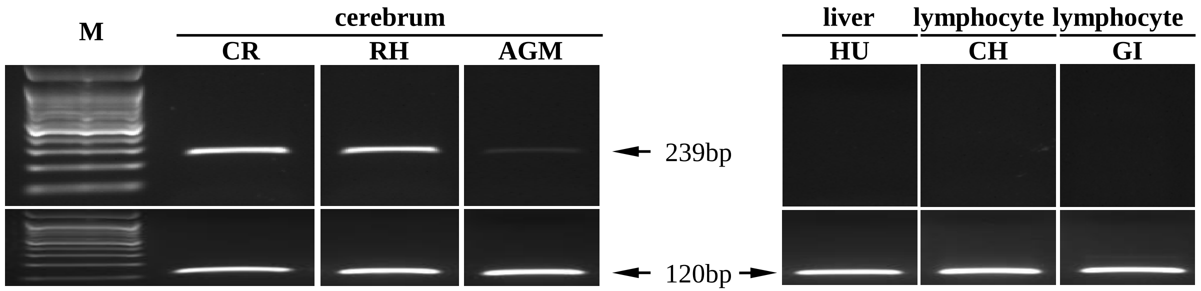

HU: human, CH: chimpanzee, GI: gibbon, RH: rhesus monkey, CR: crab-eating monkey, AGM: African green monkey, M: Marker

Figure S5. RT-PCR amplification for validation of the V2 transcript using Primer 3. A 239 bp product of the V2 transcript was detected in the crab-eating monkey, rhesus monkey, and African green monkey and was not detected in human, chimpanzee, and gibbon samples.

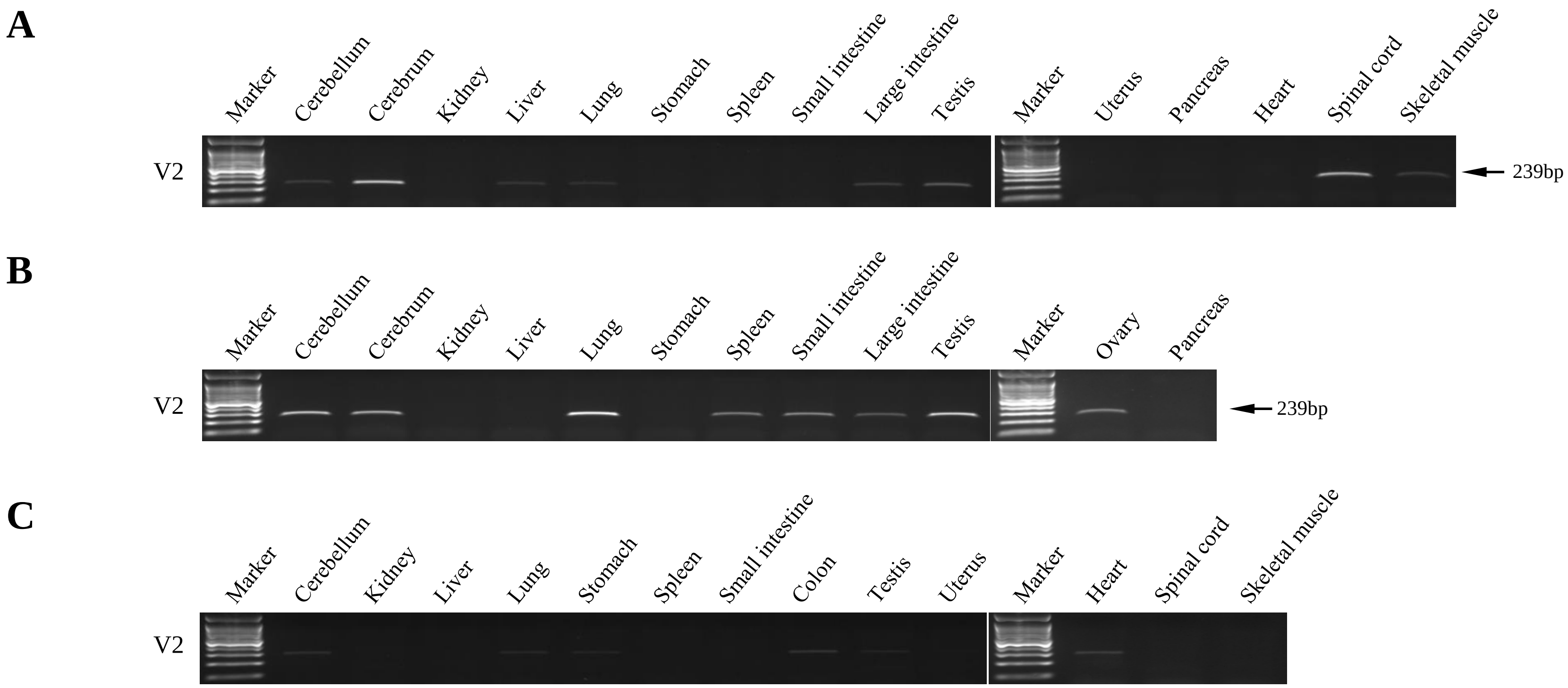

Figure S6. RT-PCR amplification for expression pattern of V2 transcript (239 bp) in the crab-eating monkey (A), rhesus monkey (B), and human (C). The experiments were performed with 35 cycles of 94 °C for 30 s, 59 °C for 30 s, and 72 °C for 30 s because 30 cycles of V2 PCR amplification in Fig. 4 doesn′t show clear target bands.

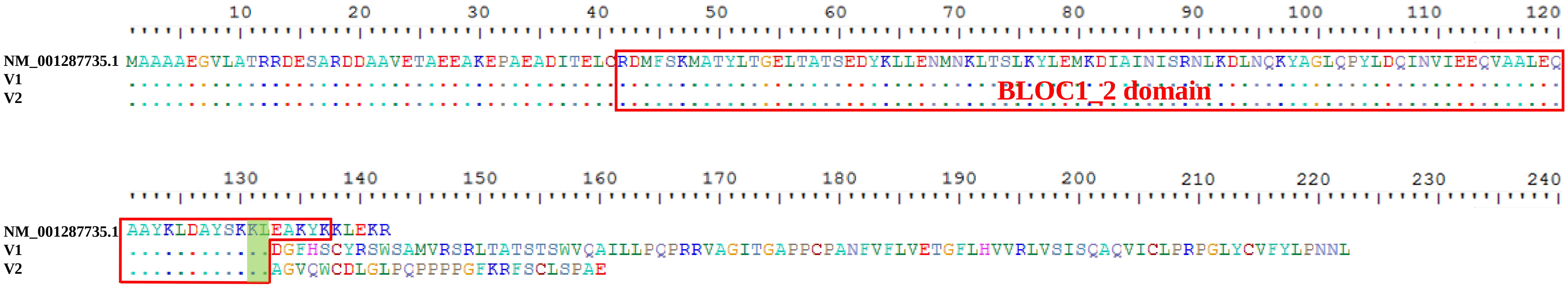

Figure S7. Multiple alignment of the amino acid sequences of the reference transcript, V1, and V2 transcripts of *BLOC1S2*. Lysine (K), Leucine (L) in green rectangular part are equivalent to the last part of the 4th exon nucleotide sequences of *BLOC1S2* transcript and see Fig. 5 for more details.

Table 2. Calculation of MIR_*Alu*Sp combination throughout the genome

	Transposable element	Crab-eating monkey	Rhesus monkey	Human
A	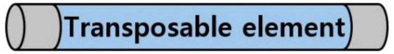	**4,325,033**	**4,725,623**	**4,687,717**
B	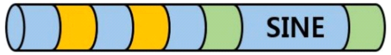	**1,754,137** (41%)*	**1,900,616** (40%)*	**1,852,545** (40%)*
C	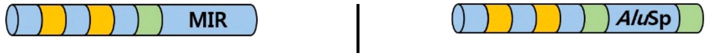	**163,269** (4%)* | **46,822** (1%)*	**170,375** (4%)* | **63,623** (1%)*	**176,635** (4%)* |**53,809** (1%)*
D	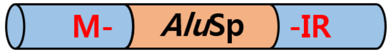	**209**	**262**	**252**
E	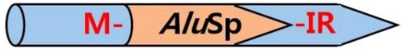	**125**	**166**	**145**
F	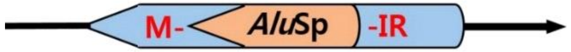 **	**4**	**15**	**29**
G	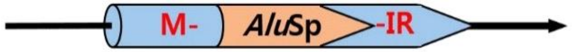	**0**	**8**	**33**

A : Total number of TEs in each genome**;** **B** : Total number of SINEs in each genome; **C**_left : Total number of *Alu*Sp in each genome; **C**_right : Total number of MIR in each genome; **D** : MIR_*Alu*Sp combination; **E** : MIR_*Alu*Sp combination in the same orientation; **F** : MIR_*Alu*Sp combination with both oriented against each genome; **G** : MIR_*Alu*Sp combination with both oriented the same direction as each genome

* : Divided by total number of Transposable element

** : The same case as the analyzed MIR_*Alu*Sp orientation in this study
